# Supplementary material for: The Retinal Dopaminergic Circuit as a Biomarker for Huntington’s and Alzheimer’s Diseases
Source: Int J Mol Sci. 2025 Jun 10;26(12):5532. doi: 10.3390/ijms26125532 (PMC12193662; doi:10.3390/ijms26125532)
Supplement: Supplementary file 1 [file ijms-26-05532-s001.zip › ijms-3575768-supplementary.pdf]

Supplementary Figure S1

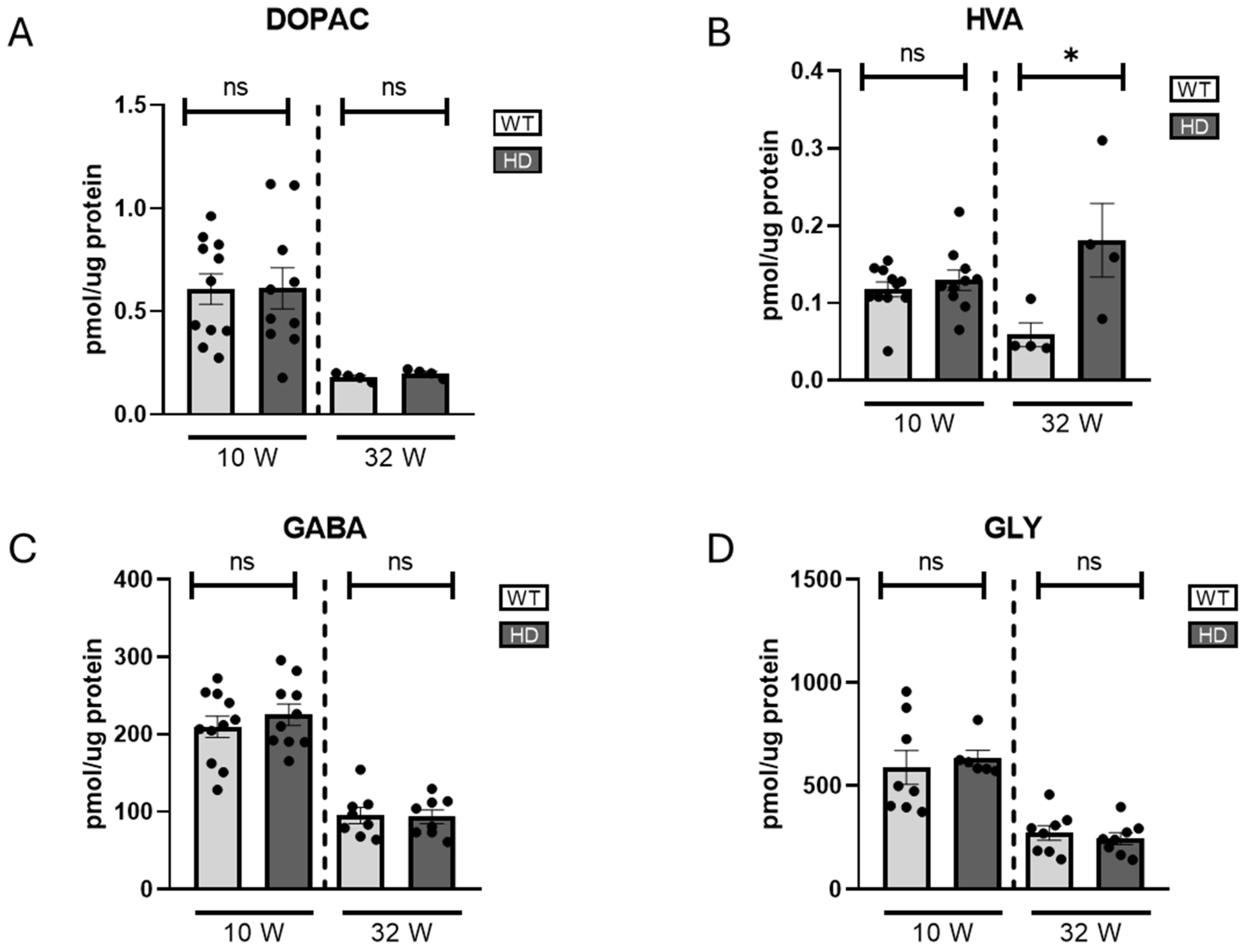

S1. Neurotransmitters levels and DA degradation metabolites measured in HD mice retina. (**A**) Homovanillic acid (HVA), (**B**) 3,4-dihydroxyphenylacetic acid (DOPAC), (**C**) GABA and (**D**) GLY were measured by HPLC at 10-and 32 weeks old retinas from WT or HD mice. Ns= non-significant, *=p<0.05.

Supplementary Figure S2

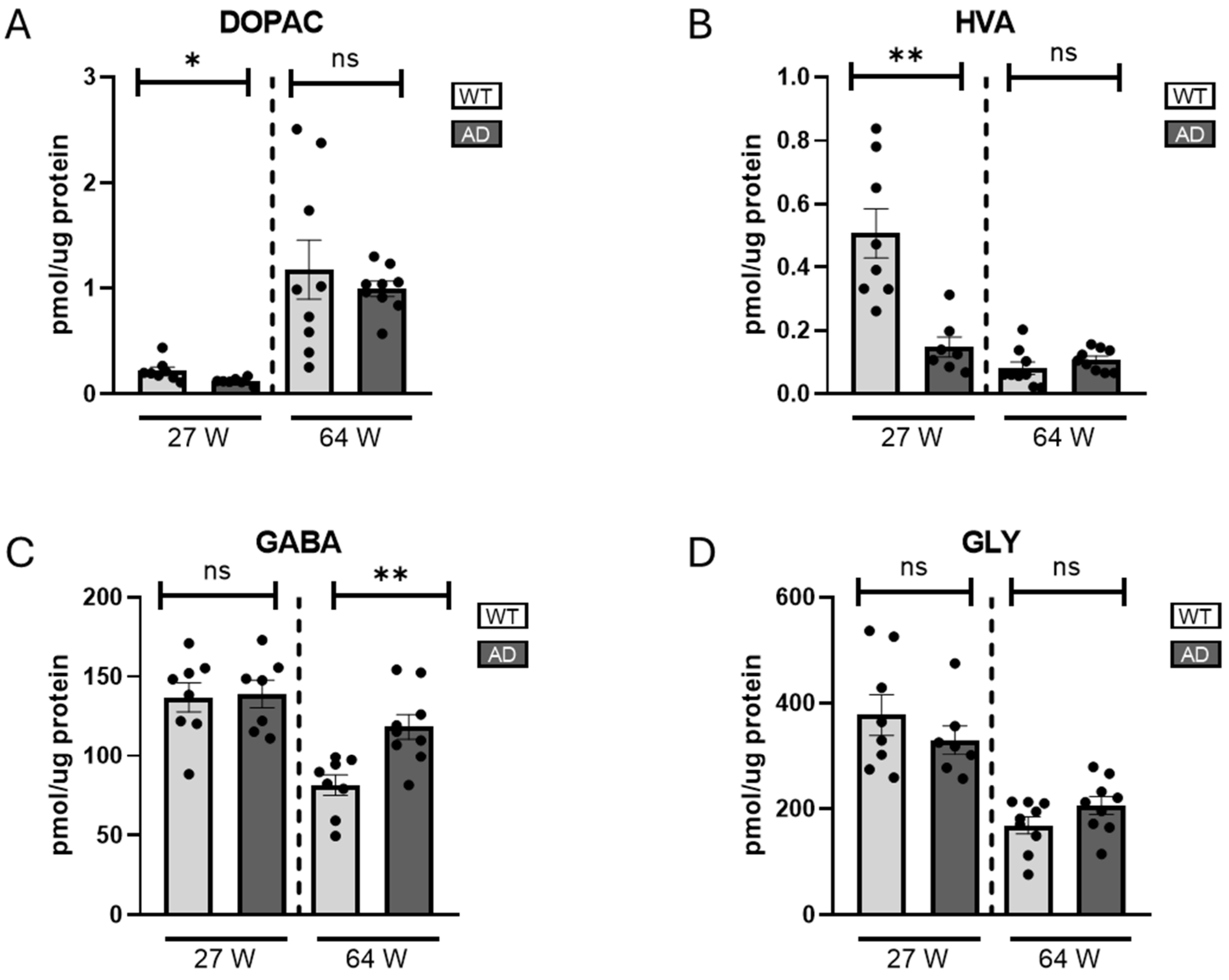

S2. Neurotransmitters levels and DA degradation metabolites in AD mice retina. (**A**) Homovanillic acid (HVA), (**B**) 3,4-dihydroxyphenylacetic acid (DOPAC), (**C**) GABA and (**D**) GLY were measured by HPLC a t 27-and 64 weeks old retinas from WT or AD mice. ns=non-significant, *=p<0.05, **= p<0.01

Supplementary Figure S3

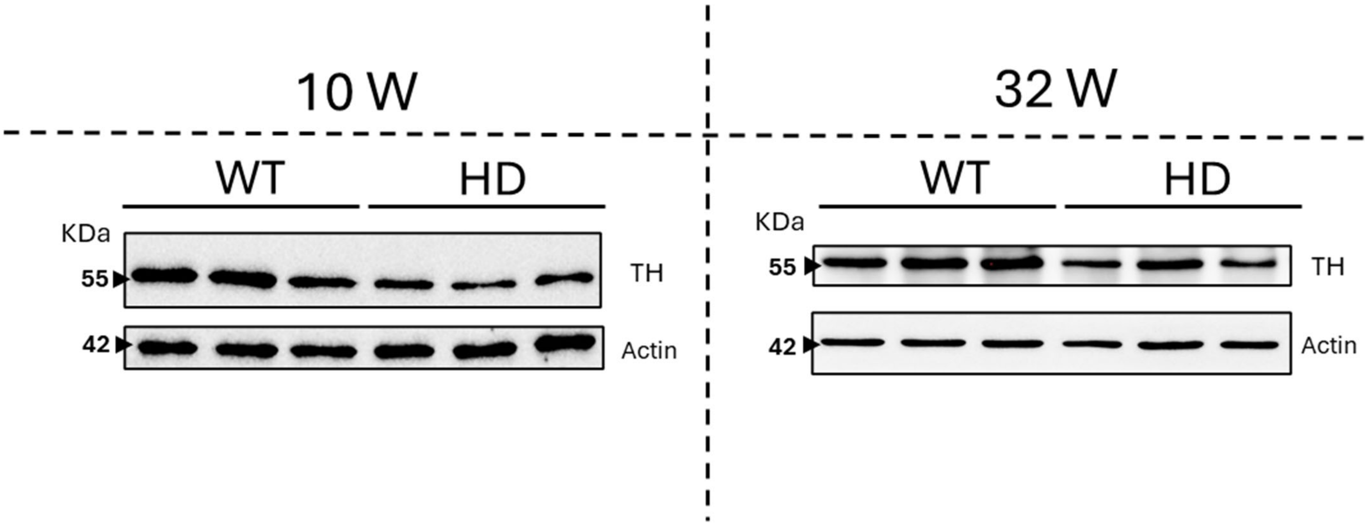

S3. Representative Western Blot from HD mice. Protein extracts from the retina of R6/1 mice and WT mice were analyzed by western blotting assays with antibodies against Tyrosine Hydroxylase (TH) and actin as loading control of 10-weeks old mice and 32-weeks old mice (n=3-11).

Supplementary Figure S4

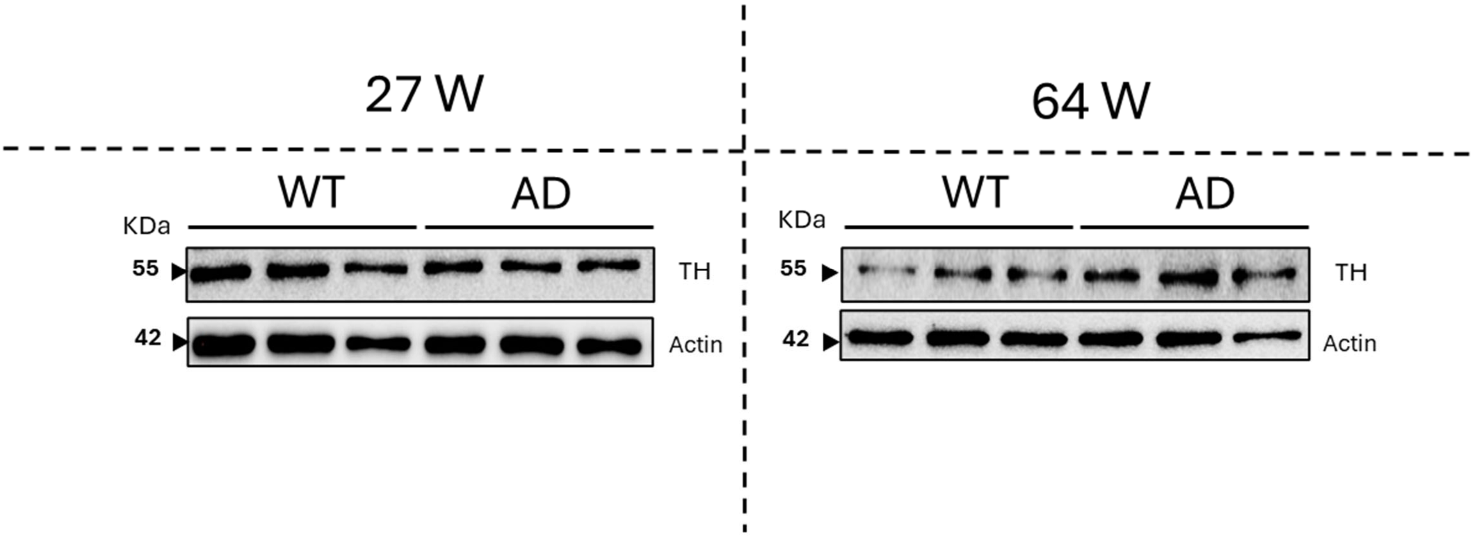

S4. Representative Western Blot from AD mice retinas. Protein extracts from the retina of APP/PSEN1 mice and WT mice were analysed by western blotting assays with antibodies against Tyrosine Hydroxylase (TH) and actin as loading control of 27-weeks old mice and 64-weeks old mice (n=3-11).

Supplementary Table S1: Retinal and visual defects in patients with Huntington's disease.

Methodology	Phenotype	References
**OCT**	ONL thickness increased.<br>Reduction in total retina.<br>Infra-, superior- and temporal RNFL thickness reduced.	**[1–4]**
	pRNFL thicker in pre-HD patients and thinner in HD patients.<br>Decreased ELM-BM in pre-HD and HD patients.	**[5,6]**
	TCF stage III. Decreased RNFL.	**[2]**
	Reduced macular volume	**[7]**
**ERG**	Increase in photopic pathway	**[8]**
	Decrease in mfERG and ffERG	**[9]**
**Visual acuity tests**	Colour contrast deficiency	**[1,3,10]**
	Deficit in forms contrast sensitivity	**[11]**

[1]Optical Coherence Tomography (OCT), Electroretinogram (ERG), Outer Nuclear Layer (ONL), Retinal Fiber Nerve Later (RNFL), Peripapillary Retinal Fiber Nerve Layer (pRNFL), External Limiting Membrane-Brunch's Membrane complex (ELM-BM), Total Functional Capacity (TCF), multifocal ERG (mfERG), full field ERG (ffERG)).

Supplementary Table S2: Retinal and visual impairments in Huntington's disease mice models

Methodology	Phenotype	Reference
**ERG**	Decreased in photopic ffERG	**[12]**
	Latency of a-wave in photopic condition increased<br>Total depletion of b-wave in photopic condition and no pERG	**[13]**
	Photopic ffERG lowered	**[14]**
	Decline in a and b-waves in scotopic condition<br>Decrease of b-wave in photopic conditions	**[15]**
	Cone response decreased	**[16]**
**Histology**	mHTT inclusions in GCL<br>Wavy ONL<br>Disruption and shortening of OS<br>Vacuolization of photoreceptors in OS<br>Cones lost<br>Photoreceptor cilium transport	**[12,14,15,17–21]**
	Abnormal dendrites of bipolar cells<br>Loss of photoreceptors<br>Abnormal synapsis in OPL<br>Loss of RGC and abnormal optic nerve structure	**[15,16,22]**
	Reduced melanopsin expression	**[18]**
	Death of ipRGCs	**[23]**
**Visual acuity tests**	Decline in visual acuity	**[15]**
	Reduced pupil reflex	**[18]**
	A1 astrocyte polarization	**[20]**

[2]Electroretinogram (ERG), full field ERG (ffERG), pattern ERG (pERG), mutant huntingtin (mHTT), Outer Nuclear Layer (ONL), Outer Segments (OS), Outer Plexiform Layer (OPL), Retinal Ganglion Cell (RGC), intrinsically photosensitive RGC (ipRGC).

Supplementary Table S3: Retinal and visual defects in patients with Alzheimer's disease.

Methodology	Phenotype	Reference
**OCT**	Decreased macular volume.	**[24]**
	Thinning in pRNFL.	**[25–27]**
	Thinning of total RNFL<br>Reduced macular volume in AD but increased in MCI.<br>Thinning in all the retina layers and ONL.	**[28–37]**
	Reduced GCL-IPL thickness.	**[31]**
	Thicker GCL-IPL volume in APOε4 carriers.	**[38]**
**ERG**	Suppression of mfERG<br>Contrast sensitivity altered	**[28,37]**
	Reduced pERG	**[39]**
**Histology**	Identification of Aβ-plaques in the retina and loss of RGC	**[40–44]**
	Loss of ipRGC cells, thinner dendrites and intracellular Aβ deposits	**[32]**
	Presence of Aβ oligomers and Aβ deposits	**[45]**
	p-TAU deposits in IPL and OPL and intracellular deposits in INL and ONL<br>p-TAU deposits in horizontal and amacrine cells	**[41,46–48]**
	Deposition of non-phosphorylated Tau in GCL	**[49]**
**Visual acuity tests**	Spatial contrast impaired	**[33,36]**
	Colour contrast deficiency related with decreased macular volume and RNFL thinning	
	Ishihara tests low scores show colour contrast deficit and can be used as screening	**[50]**

[3]Peripapillary Retinal Fiber Nerve Layer (pRNFL), Retinal Nerve Fiber Layer (RNFL), Mild Cognitive Impairment (MCI), Outer Nuclear Layer (ONL), Ganglion Cell Layer – Inner Plexiform Layer (GCL-IPL), Retinal Ganglion Cell (RGC), multifocal Electroretinogram (mfERG), intrinsically photosensitive RGC (ipRGC), phosphorylated Tau (p-Tau), Inner Nuclear Layer (INL), Outer Plexiform Layer (OPL).

Supplementary Table S4: Retinal and visual impairments in mice models of Alzheimer's disease.

Methodology	Phenotype	Reference
**OCT**	Thinner ONL	**[51]**
	Thinner RNFL and GCL	**[52,53]**
**ERG**	Increase a-wave latency	**[51,54]**
	Scotopic dysfunction in young mice	**[54]**
	No difference in scotopic ffERG<br>Decreased a and b-waves in photopic conditions	**[53]**
	No changes in ffERG neither pERG<br>RGC dysfunction in multielectrode array recording	**[55]**
	Lower amplitude in a-wave in photopic condition that is restored after LEB	**[56]**
	Increase a-wave of ffERG in pre-symptomatic mice	**[57]**
	ERG amplitudes decrease with age	**[58]**
	Scotopic b-wave decreased	**[59]**
	pERG is decreased	**[60]**
**Histology**	Aβ deposits	**[40,51,61,61–66]**
	Aβ deposits in GCL, IPL and INL<br>Oligomers of Aβ in GCL, INL and ONL	**[51,64,67]**
	Reduced RGC number	**[51]**
	Tau aggregation<br>Thinner RNFL and GCL	**[52,53]**
	RGC dendrites progressive loss and more dendritic varicosity<br>Retinal pathology correlates with loss of dendritic spine loss	**[68]**
	In vivo retinal imaging of fibrillar tau<br>p-tau tangles	**[69]**
**Visual acuity tests**	Reduced visual acuity	**[55]**
	Colour contrast deficit	**[70]**

[4]Ganglion Cell Layer (GCL), Inner Plexiform Layer (IPL), Inner Nuclear Layer (INL), Outer Nuclear Layer (ONL), Retinal Ganglion Cell (RGC), intrinsically photosensitive RGC (ipRGC), Retinal Nerve Fiber Layer (RNFL), phosphorylated-Tau (p-Tau), Electroretinogram (ERG), full field ERG (ffERG), pattern ERG (pERG), Inmunohistochemistry (IHC).

References

1. Kersten, H.M.; Danesh-Meyer, H.V.; Kilfoyle, D.H.; Roxburgh, R.H. Optical Coherence Tomography Findings in Huntington's Disease: A Potential Biomarker of Disease Progression. *J Neurol* 2015, *262*, 2457–2465, doi:10.1007/s00415-015-7869-2.

2. Gatto, E.; Parisi, V.; Persi, G.; Fernandez Rey, E.; Cesarini, M.; Luis Etcheverry, J.; Rivera, P.; Squitieri, F. Optical Coherence Tomography (OCT) Study in Argentinean Huntington's Disease Patients. *International Journal of Neuroscience* 2018, *128*, 1157–1162, doi:10.1080/00207454.2018.1489807.

3. Gulmez Sevim, D.; Unlu, M.; Gultekin, M.; Karaca, C. Retinal Single-Layer Analysis with Optical Coherence Tomography Shows Inner Retinal Layer Thinning in Huntington's Disease as a Potential Biomarker. *Int Ophthalmol* 2019, *39*, 611–621, doi:10.1007/s10792-018-0857-7.

4. Mazur-Michałek, I.; Kowalska, K.; Zielonka, D.; Leśniczak-Staszak, M.; Pietras, P.; Szaflarski, W.; Isalan, M.; Mielcarek, M. Structural Abnormalities of the Optic Nerve and Retina in Huntington's Disease Pre-Clinical and Clinical Settings. *Int J Mol Sci* 2022, *23*, 5450, doi:10.3390/ijms23105450.

5. Amini, E.; Moghaddasi, M.; Habibi, S.A.H.; Azad, Z.; Miri, S.; Nilforushan, N.; Mirshahi, R.; Cubo, E.; Mohammadzadeh, N.; Rohani, M. Huntington's Disease and Neurovascular Structure of Retina. *Neurol Sci* 2022, *43*, 5933–5941, doi:10.1007/s10072-022-06232-3.

6. Murueta-Goyena, A.; Del Pino, R.; Acera, M.; Teijeira-Portas, S.; Romero, D.; Ayala, U.; Fernández-Valle, T.; Tijero, B.; Gabilondo, I.; Gómez Esteban, J.C. Retinal Thickness as a Biomarker of Cognitive Impairment in Manifest Huntington's Disease. *J Neurol* 2023, *270*, 3821–3829, doi:10.1007/s00415-023-11720-3.

7. Haider, S.; Raftopoulos, R.; Kapoor, R.; Tabrizi, S.J. E29 Macular Volume Loss In Huntington's Disease On Optical Coherence Tomography- A Pilot Biomarker Study. *J Neurol Neurosurg Psychiatry* 2014, *85*, A46–A47, doi:10.1136/jnnp-2014-309032.132.

8. Pearl, J.R.; Heath, L.M.; Bergey, D.E.; Kelly, J.P.; Smith, C.; Laurino, M.Y.; Weiss, A.; Price, N.D.; LaSpada, A.; Bird, T.D.; et al. Enhanced Retinal Responses in Huntington's Disease Patients. *J Huntingtons Dis* 2017, *6*, 237–247, doi:10.3233/JHD-170255.

9. Knapp, J.; VanNasdale, D.A.; Ramsey, K.; Racine, J. Retinal Dysfunction in a Presymptomatic Patient with Huntington's Disease. *Doc Ophthalmol* 2018, *136*, 213–221, doi:10.1007/s10633-018-9632-3.

10. Büttner, Th.; Schulz, S.; Kuhn, W.; Blumenschein, A.; Przuntek, H. Impaired Colour Discrimination in Huntington's Disease. *European Journal of Neurology* 1994, *1*, 153–157, doi:10.1111/j.1468-1331.1994.tb00064.x.

11. O'Donnel, B.F.; BLEKHER, T.M.; WEAVER, M.; WHITE, K.M.; MARSHALL, J.; BERISTAIN, X.; STOUT, J.C.; GRAY, J.; WOJCIESZEK, J.M.; FOROUD, T.M. Visual Perception in Prediagnostic and Early Stage Huntington's Disease. *J Int Neuropsychol Soc* 2008, *14*, 446–453, doi:10.1017/S1355617708080405.

12. Helmlinger, D.; Yvert, G.; Picaud, S.; Merienne, K.; Sahel, J.; Mandel, J.-L.; Devys, D. Progressive Retinal Degeneration and Dysfunction in R6 Huntington's Disease Mice. *Human Molecular Genetics* 2002, *11*, 3351–3359, doi:10.1093/hmg/11.26.3351.

13. Ragauskas, S.; Leinonen, H.; Puranen, J.; Rönkkö, S.; Nymark, S.; Gurevicius, K.; Lipponen, A.; Kontkanen, O.; Puoliväli, J.; Tanila, H.; et al. Early Retinal Function Deficit without Prominent Morphological Changes in the R6/2 Mouse Model of Huntington's Disease. *PLoS One* 2014, *9*, e113317, doi:10.1371/journal.pone.0113317.

14. Li, M.; Yasumura, D.; Ma, A.A.K.; Matthes, M.T.; Yang, H.; Nielson, G.; Huang, Y.; Szoka, F.C.; LaVail, M.M.; Diamond, M.I. Intravitreal Administration of HA-1077, a ROCK Inhibitor, Improves Retinal Function in a Mouse Model of Huntington Disease. *PLoS One* 2013, *8*, e56026, doi:10.1371/journal.pone.0056026.

15. Yang, D.; Huang, C.; Guo, X.; Li, Y.; Wu, J.; Zhang, Z.; Yan, S.; Xu, Y. Abnormal Outer and Inner Retina in a Mouse Model of Huntington's Disease with Age. *Front Aging Neurosci* 2024, *16*, 1434551, doi:10.3389/fnagi.2024.1434551.

16. Batcha, A.H.; Greferath, U.; Jobling, A.I.; Vessey, K.A.; Ward, M.M.; Nithianantharajah, J.; Hannan, A.J.; Kalloniatis, M.; Fletcher, E.L. Retinal Dysfunction, Photoreceptor Protein Dysregulation and Neuronal Remodelling in the R6/1 Mouse Model of Huntington's Disease. *Neurobiology of Disease* 2012, *45*, 887–896, doi:10.1016/j.nbd.2011.12.004.

17. Karam, A.; Tebbe, L.; Weber, C.; Messaddeq, N.; Morlé, L.; Kessler, P.; Wolfrum, U.; Trottier, Y. A Novel Function of Huntingtin in the Cilium and Retinal Ciliopathy in Huntington's Disease Mice. *Neurobiology of Disease* 2015, *80*, 15–28, doi:10.1016/j.nbd.2015.05.008.

18. Ouk, K.; Hughes, S.; Pothecary, C.A.; Peirson, S.N.; Morton, A.J. Attenuated Pupillary Light Responses and Downregulation of Opsin Expression Parallel Decline in Circadian Disruption in Two Different Mouse Models of Huntington's Disease. *Hum Mol Genet* 2016, *25*, 5418–5432, doi:10.1093/hmg/ddw359.

19. Yefimova, M.G.; Béré, E.; Cantereau-Becq, A.; Meunier-Balandre, A.-C.; Merceron, B.; Burel, A.; Merienne, K.; Ravel, C.; Becq, F.; Bourmeyster, N. Myelinosome Organelles in the Retina of R6/1 Huntington Disease (HD) Mice: Ubiquitous Distribution and Possible Role in Disease Spreading. *Int J Mol Sci* 2021, *22*, 12771, doi:10.3390/ijms222312771.

20. Cano-Cano, F.; Martín-Loro, F.; Gallardo-Orihuela, A.; González-Montelongo, M. del C.; Ortuño-Miquel, S.; Hervás-Corpión, I.; de la Villa, P.; Ramón-Marco, L.; Navarro-Calvo, J.; Gómez-Jaramillo, L.; et al. Retinal Dysfunction in Huntington's Disease Mouse Models Concurs with Local Gliosis and Microglia Activation. *Sci Rep* 2024, *14*, 4176, doi:10.1038/s41598-024-54347-8.

21. Xu, H.; Ajayan, A.; Langen, R.; Chen, J. Pleiotropic Effects of Mutant Huntingtin on Retinopathy in Two Mouse Models of Huntington's Disease. *Neurobiol Dis* 2025, *205*, 106780, doi:10.1016/j.nbd.2024.106780.

22. Petrasch-Parwez, E.; Habbes, H.; Weickert, S.; Löbbecke-Schumacher, M.; Striedinger, K.; Wieczorek, S.; Dermietzel, R.; Epplen, J.T. Fine-structural Analysis and Connexin Expression in the Retina of a Transgenic Model of Huntington's Disease. *J of Comparative Neurology* 2004, *479*, 181–197, doi:10.1002/cne.20327.

23. Lin, M.-S.; Liao, P.-Y.; Chen, H.-M.; Chang, C.-P.; Chen, S.-K.; Chern, Y. Degeneration of ipRGCs in Mouse Models of Huntington's Disease Disrupts Non-Image-Forming Behaviors Before Motor Impairment. *J Neurosci* 2019, *39*, 1505–1524, doi:10.1523/JNEUROSCI.0571-18.2018.

24. Byun, M.S.; Park, S.W.; Lee, J.H.; Yi, D.; Jeon, S.Y.; Choi, H.J.; Joung, H.; Ghim, U.H.; Park, U.C.; Kim, Y.K.; et al. Association of Retinal Changes With Alzheimer Disease Neuroimaging Biomarkers in Cognitively Normal Individuals. *JAMA Ophthalmol* 2021, *139*, 548–556, doi:10.1001/jamaophthalmol.2021.0320.

25. Lu, Y.; Li, Z.; Zhang, X.; Ming, B.; Jia, J.; Wang, R.; Ma, D. Retinal Nerve Fiber Layer Structure Abnormalities in Early Alzheimer's Disease: Evidence in Optical Coherence Tomography. *Neuroscience Letters* 2010, *480*, 69–72, doi:10.1016/j.neulet.2010.06.006.

26. Cunha, J.P.; Proença, R.; Dias-Santos, A.; Almeida, R.; Águas, H.; Alves, M.; Papoila, A.L.; Louro, C.; Castanheira-Dinis, A. OCT in Alzheimer's Disease: Thinning of the RNFL and Superior Hemiretina. *Graefes Arch Clin Exp Ophthalmol* 2017, *255*, 1827–1835, doi:10.1007/s00417-017-3715-9.

27. Ferrari, L.; Huang, S.-C.; Magnani, G.; Ambrosi, A.; Comi, G.; Leocani, L. Optical Coherence Tomography Reveals Retinal Neuroaxonal Thinning in Frontotemporal Dementia as in Alzheimer's Disease. *Journal of Alzheimer's Disease* 2017, *56*, 1101–1107, doi:10.3233/JAD-160886.

28. Moschos, M.M.; Markopoulos, I.; Chatziralli, I.; Rouvas, A.; Papageorgiou, S.G.; Ladas, I.; Vassilopoulos, D. Structural and Functional Impairment of the Retina and Optic Nerve in Alzheimer's Disease. *Current Alzheimer Research* 2012, *9*, 782–788, doi:10.2174/156720512802455340.

29. Ascaso, F.J.; Cruz, N.; Modrego, P.J.; Lopez-Anton, R.; Santabárbara, J.; Pascual, L.F.; Lobo, A.; Cristóbal, J.A. Retinal Alterations in Mild Cognitive Impairment and Alzheimer's Disease: An Optical Coherence Tomography Study. *J Neurol* 2014, *261*, 1522–1530, doi:10.1007/s00415-014-7374-z.

30. Kromer, R.; Serbecic, N.; Hausner, L.; Aboul-Enein, F.; Froelich, L.; Beutelspacher, S. Detection of Retinal Nerve Fiber Layer Defects in Alzheimer's Disease Using SD-OCT. *Front. Psychiatry* 2014, *5*, doi:10.3389/fpsyt.2014.00022.

31. Cheung, C.Y.; Ong, Y.T.; Hilal, S.; Ikram, M.K.; Low, S.; Ong, Y.L.; Venketasubramanian, N.; Yap, P.; Seow, D.; Chen, C.L.H.; et al. Retinal Ganglion Cell Analysis Using High-Definition Optical Coherence Tomography in Patients with Mild Cognitive Impairment and Alzheimer's Disease. *Journal of Alzheimer's Disease* 2015, *45*, 45–56, doi:10.3233/JAD-141659.

32. La Morgia, C.; Ross-Cisneros, F.N.; Koronyo, Y.; Hannibal, J.; Gallassi, R.; Cantalupo, G.; Sambati, L.; Pan, B.X.; Tozer, K.R.; Barboni, P.; et al. Melanopsin Retinal Ganglion Cell Loss in Alzheimer Disease. *Annals of Neurology* 2016, *79*, 90–109, doi:10.1002/ana.24548.

33. Polo, V.; Rodrigo, M.J.; Garcia-Martin, E.; Otin, S.; Larrosa, J.M.; Fuertes, M.I.; Bambo, M.P.; Pablo, L.E.; Satue, M. Visual Dysfunction and Its Correlation with Retinal Changes in Patients with Alzheimer's Disease. *Eye* 2017, *31*, 1034–1041, doi:10.1038/eye.2017.23.

34. Ko, F.; Muthy, Z.A.; Gallacher, J.; Sudlow, C.; Rees, G.; Yang, Q.; Keane, P.A.; Petzold, A.; Khaw, P.T.; Reisman, C.; et al. Association of Retinal Nerve Fiber Layer Thinning With Current and Future Cognitive Decline: A Study Using Optical Coherence Tomography. *JAMA Neurology* 2018, *75*, 1198–1205, doi:10.1001/jamaneurol.2018.1578.

35. Jáñez-Escalada, L.; Jáñez-García, L.; Salobrar-García, E.; Santos-Mayo, A.; de Hoz, R.; Yubero, R.; Gil, P.; Ramírez, J.M. Spatial Analysis of Thickness Changes in Ten Retinal Layers of Alzheimer's Disease Patients Based on Optical Coherence Tomography. *Scientific Reports* 2019, *9*, doi:10.1038/s41598-019-49353-0.

36. Salobrar-García, E.; Hoz, R. de; Ramírez, A.I.; López-Cuenca, I.; Rojas, P.; Vazirani, R.; Amarante, C.; Yubero, R.; Gil, P.; Pinazo-Durán, M.D.; et al. Changes in Visual Function and Retinal Structure in the Progression of Alzheimer's Disease. *PLOS ONE* 2019, *14*, e0220535, doi:10.1371/journal.pone.0220535.

37. Sen, S.; Saxena, R.; Vibha, D.; Tripathi, M.; Sharma, P.; Phuljhele, S.; Tandon, R.; Kumar, P. Detection of Structural and Electrical Disturbances in Macula and Optic Nerve in Alzheimer's Patients and Their Correlation with Disease Severity. *Seminars in Ophthalmology* 2020, *35*, 116–125, doi:10.1080/08820538.2020.1748203.

38. Rotenstreich, Y.; Sharvit-Ginon, I.; Sher, I.; Zloto, O.; Fabian, I.D.; Abd-Elkader, A.; Weller, A.; Heymann, A.; Beeri, M.S.; Ravona-Springer, R. Thicker Macula in Asymptomatic APOE Ɛ4 Middle-aged Adults at High AD Risk. *Alzheimers Dement (Amst)* 2022, *14*, e12275, doi:10.1002/dad2.12275.

39. Parisi, V. Correlation between Morphological and Functional Retinal Impairment in Patients Affected by Ocular Hypertension, Glaucoma, Demyelinating Optic Neuritis and Alzheimer's Disease. *Seminars in Ophthalmology* 2003, *18*, 50–57, doi:10.1076/soph.18.2.50.15855.

40. Koronyo-Hamaoui, M.; Koronyo, Y.; Ljubimov, A.V.; Miller, C.A.; Ko, M.K.; Black, K.L.; Schwartz, M.; Farkas, D.L. Identification of Amyloid Plaques in Retinas from Alzheimer's Patients and Noninvasive *in Vivo* Optical Imaging of Retinal Plaques in a Mouse Model. *NeuroImage* 2011, *54*, S204–S217, doi:10.1016/j.neuroimage.2010.06.020.

41. den Haan, J.; Morrema, T.H.J.; Verbraak, F.D.; de Boer, J.F.; Scheltens, P.; Rozemuller, A.J.; Bergen, A.A.B.; Bouwman, F.H.; Hoozemans, J.J. Amyloid-Beta and Phosphorylated Tau in Post-Mortem Alzheimer's Disease Retinas. *Acta Neuropathol Commun* 2018, *6*, 147, doi:10.1186/s40478-018-0650-x.

42. Shi, H.; Koronyo, Y.; Rentsendorj, A.; Regis, G.C.; Sheyn, J.; Fuchs, D.-T.; Kramerov, A.A.; Ljubimov, A.V.; Dumitrascu, O.M.; Rodriguez, A.R.; et al. Identification of Early Pericyte Loss and Vascular Amyloidosis in Alzheimer's Disease Retina. *Acta Neuropathol* 2020, *139*, 813–836, doi:10.1007/s00401-020-02134-w.

43. Liao, C.; Xu, J.; Chen, Y.; Ip, N.Y. Retinal Dysfunction in Alzheimer's Disease and Implications for Biomarkers. *Biomolecules* 2021, *11*, 1215, doi:10.3390/biom11081215.

44. García-Bermúdez, M.Y.; Vohra, R.; Freude, K.; Wijngaarden, P. van; Martin, K.; Thomsen, M.S.; Aldana, B.I.; Kolko, M. Potential Retinal Biomarkers in Alzheimer's Disease. *International Journal of Molecular Sciences* 2023, *24*, 15834, doi:10.3390/ijms242115834.

45. Koronyo, Y.; Biggs, D.; Barron, E.; Boyer, D.S.; Pearlman, J.A.; Au, W.J.; Kile, S.J.; Blanco, A.; Fuchs, D.-T.; Ashfaq, A.; et al. Retinal Amyloid Pathology and Proof-of-Concept Imaging Trial in Alzheimer's Disease. *JCI Insight* 2017, *2*, e93621, doi:10.1172/jci.insight.93621.

46. Hart de Ruyter, F.J.; Morrema, T.H.J.; den Haan, J.; Twisk, J.W.R.; de Boer, J.F.; Scheltens, P.; Boon, B.D.C.; Thal, D.R.; Rozemuller, A.J.; Verbraak, F.D.; et al. Phosphorylated Tau in the Retina Correlates with Tau Pathology in the Brain in Alzheimer's Disease and Primary Tauopathies. *Acta Neuropathol* 2023, *145*, 197–218, doi:10.1007/s00401-022-02525-1.

47. Shi, H.; Mirzaei, N.; Koronyo, Y.; Davis, M.R.; Robinson, E.; Braun, G.M.; Jallow, O.; Rentsendorj, A.; Ramanujan, V.K.; Fert-Bober, J.; et al. Identification of Retinal Tau Oligomers, Citrullinated Tau, and Other Tau Isoforms in Early and Advanced AD and Relations to Disease Status 2024, 2024.02.13.579999.

48. Walkiewicz, G.; Ronisz, A.; Van Ginderdeuren, R.; Lemmens, S.; Bouwman, F.H.; Hoozemans, J.J.M.; Morrema, T.H.J.; Rozemuller, A.J.; Hart de Ruyter, F.J.; De Groef, L.; et al. Primary Retinal Tauopathy: A Tauopathy with a Distinct Molecular Pattern. *Alzheimer's & Dementia* 2024, *20*, 330–340, doi:10.1002/alz.13424.

49. Leger, F.; Fernagut, P.-O.; Canron, M.-H.; Léoni, S.; Vital, C.; Tison, F.; Bezard, E.; Vital, A. Protein Aggregation in the Aging Retina. *Journal of Neuropathology & Experimental Neurology* 2011, *70*, 63–68, doi:10.1097/NEN.0b013e31820376cc.

50. Kim, H.J.; Ryou, J.H.; Choi, K.T.; Kim, S.M.; Kim, J.T.; Han, D.H. Deficits in Color Detection in Patients with Alzheimer Disease. *PLoS One* 2022, *17*, e0262226, doi:10.1371/journal.pone.0262226.

51. Vandenabeele, M.; Veys, L.; Lemmens, S.; Hadoux, X.; Gelders, G.; Masin, L.; Serneels, L.; Theunis, J.; Saito, T.; Saido, T.C.; et al. The AppNL-G-F Mouse Retina Is a Site for Preclinical Alzheimer's Disease Diagnosis and Research. *Acta Neuropathol Commun* 2021, *9*, 6, doi:10.1186/s40478-020-01102-5.

52. Buccarello, L.; Sclip, A.; Sacchi, M.; Castaldo, A.M.; Bertani, I.; ReCecconi, A.; Maestroni, S.; Zerbini, G.; Nucci, P.; Borsello, T. The C-Jun N-Terminal Kinase Plays a Key Role in Ocular Degenerative Changes in a Mouse Model of Alzheimer Disease Suggesting a Correlation between Ocular and Brain Pathologies. *Oncotarget* 2017, *8*, 83038–83051, doi:10.18632/oncotarget.19886.

53. Wang, S.; Jiang, X.; Peng, W.; Yang, S.; Pi, R.; Zhou, S. Acrolein Induces Retinal Abnormalities of Alzheimer's Disease in Mice. *International Journal of Molecular Sciences* 2023, *24*, 13576, doi:10.3390/ijms241713576.

54. McAnany, J.J.; Matei, N.; Chen, Y.-F.; Liu, K.; Park, J.C.; Shahidi, M. Rod Pathway and Cone Pathway Retinal Dysfunction in the 5xFAD Mouse Model of Alzheimer's Disease. *Sci Rep* 2021, *11*, 4824, doi:10.1038/s41598-021-84318-2.

55. Zhang, M.; Zhong, L.; Han, X.; Xiong, G.; Xu, D.; Zhang, S.; Cheng, H.; Chiu, K.; Xu, Y. Brain and Retinal Abnormalities in the 5xFAD Mouse Model of Alzheimer's Disease at Early Stages. *Front Neurosci* 2021, *15*, 681831, doi:10.3389/fnins.2021.681831.

56. Sun, Z.; Liu, J.; Chen, Z.; So, K.; Hu, Y.; Chiu, K. Lycium Barbarum Extract Enhanced Neuroplasticity and Functional Recovery in 5xFAD Mice via Modulating Microglial Status of the Central Nervous System. *CNS Neurosci Ther* 2024, *30*, e70123, doi:10.1111/cns.70123.

57. Dinet, V.; Arouche-Delaperche, L.; Dégardin, J.; Naud, M.-C.; Picaud, S.; Krantic, S. Concomitant Retinal Alterations in Neuronal Activity and TNFα Pathway Are Detectable during the Pre-Symptomatic Stage in a Mouse Model of Alzheimer's Disease. *Cells* 2022, *11*, 1650, doi:10.3390/cells11101650.

58. Liu, J.; Baum, L.; Yu, S.; Lin, Y.; Xiong, G.; Chang, R.C.-C.; So, K.F.; Chiu, K. Preservation of Retinal Function Through Synaptic Stabilization in Alzheimer's Disease Model Mouse Retina by Lycium Barbarum Extracts. *Front Aging Neurosci* 2022, *13*, 788798, doi:10.3389/fnagi.2021.788798.

59. Do, K.V.; Kautzmann, M.-A.I.; Jun, B.; Gordon, W.C.; Nshimiyimana, R.; Yang, R.; Petasis, N.A.; Bazan, N.G. Elovanoids Counteract Oligomeric β-Amyloid-Induced Gene Expression and Protect Photoreceptors. *Proc Natl Acad Sci U S A* 2019, *116*, 24317–24325, doi:10.1073/pnas.1912959116.

60. Frame, G.; Schuller, A.; Smith, M.A.; Crish, S.D.; Dengler-Crish, C.M. Alterations in Retinal Signaling Across Age and Sex in 3xTg Alzheimer's Disease Mice. *J Alzheimers Dis* 2022, *88*, 471–492, doi:10.3233/JAD-220016.

61. Grimaldi, A.; Brighi, C.; Peruzzi, G.; Ragozzino, D.; Bonanni, V.; Limatola, C.; Ruocco, G.; Di Angelantonio, S. Inflammation, Neurodegeneration and Protein Aggregation in the Retina as Ocular Biomarkers for Alzheimer's Disease in the 3xTg-AD Mouse Model. *Cell Death Dis* 2018, *9*, 685, doi:10.1038/s41419-018-0740-5.

62. Hadoux, X.; Hui, F.; Lim, J.K.H.; Masters, C.L.; Pébay, A.; Chevalier, S.; Ha, J.; Loi, S.; Fowler, C.J.; Rowe, C.; et al. Non-Invasive in Vivo Hyperspectral Imaging of the Retina for Potential Biomarker Use in Alzheimer's Disease. *Nat Commun* 2019, *10*, 4227, doi:10.1038/s41467-019-12242-1.

63. Doustar, J.; Rentsendorj, A.; Torbati, T.; Regis, G.C.; Fuchs, D.; Sheyn, J.; Mirzaei, N.; Graham, S.L.; Shah, P.K.; Mastali, M.; et al. Parallels between Retinal and Brain Pathology and Response to Immunotherapy in Old, Late-stage Alzheimer's Disease Mouse Models. *Aging Cell* 2020, *19*, e13246, doi:10.1111/acel.13246.

64. Habiba, U.; Merlin, S.; Lim, J.K.H.; Wong, V.H.Y.; Nguyen, C.T.O.; Morley, J.W.; Bui, B.V.; Tayebi, M. Age-Specific Retinal and Cerebral Immunodetection of Amyloid-β Plaques and Oligomers in a Rodent Model of Alzheimer's Disease. *Journal of Alzheimer's Disease* 2020, *76*, 1135–1150, doi:10.3233/JAD-191346.

65. Mei, X.; Yang, M.; Zhu, L.; Zhou, Q.; Li, X.; Chen, Z.; Zou, C. Retinal Levels of Amyloid Beta Correlate with Cerebral Levels of Amyloid Beta in Young APPswe/PS1dE9 Transgenic Mice before Onset of Alzheimer's Disease. *Behav Neurol* 2020, *2020*, 1574816, doi:10.1155/2020/1574816.

66. Sidiqi, A.; Wahl, D.; Lee, S.; Ma, D.; To, E.; Cui, J.; To, E.; Beg, M.F.; Sarunic, M.; Matsubara, J.A. In Vivo Retinal Fluorescence Imaging With Curcumin in an Alzheimer Mouse Model. *Front Neurosci* 2020, *14*, 713, doi:10.3389/fnins.2020.00713.

67. Bartley, S.C.; Proctor, M.T.; Xia, H.; Ho, E.; Kang, D.S.; Schuster, K.; Bicca, M.A.; Seckler, H.S.; Viola, K.L.; Patrie, S.M.; et al. An Essential Role for Alzheimer's-Linked Amyloid Beta Oligomers in Neurodevelopment: Transient Expression of Multiple Proteoforms during Retina Histogenesis. *International Journal of Molecular Sciences* 2022, *23*, 2208, doi:10.3390/ijms23042208.

68. Bevan, R.J.; Hughes, T.R.; Williams, P.A.; Good, M.A.; Morgan, B.P.; Morgan, J.E. Retinal Ganglion Cell Degeneration Correlates with Hippocampal Spine Loss in Experimental Alzheimer's Disease. *Acta Neuropathol Commun* 2020, *8*, 216, doi:10.1186/s40478-020-01094-2.

69. Schön, C.; Hoffmann, N.A.; Ochs, S.M.; Burgold, S.; Filser, S.; Steinbach, S.; Seeliger, M.W.; Arzberger, T.; Goedert, M.; Kretzschmar, H.A.; et al. Long-Term In Vivo Imaging of Fibrillar Tau in the Retina of P301S Transgenic Mice. *PLoS One* 2012, *7*, e53547, doi:10.1371/journal.pone.0053547.

70. Vit, J.-P.; Fuchs, D.-T.; Angel, A.; Levy, A.; Lamensdorf, I.; Black, K.L.; Koronyo, Y.; Koronyo-Hamaoui, M. Color and Contrast Vision in Mouse Models of Aging and Alzheimer's Disease Using a Novel Visual-Stimuli Four-Arm Maze. *Sci Rep* 2021, *11*, 1255, doi:10.1038/s41598-021-80988-0.
